# Work-Related Exhaustion and Telomere Length: A Population-Based Study

**DOI:** 10.1371/journal.pone.0040186

**Published:** 2012-07-11

**Authors:** Kirsi Ahola, Ilari Sirén, Mika Kivimäki, Samuli Ripatti, Arpo Aromaa, Jouko Lönnqvist, Iiris Hovatta

**Affiliations:** 1 Work Organizations, Finnish Institute of Occupational Health, Helsinki, Finland; 2 Research Programs Unit – Molecular Neurology, University of Helsinki, Helsinki, Finland; 3 Department of Medical Genetics, Haartman Institute, University of Helsinki, Helsinki, Finland; 4 Department of Epidemiology and Public Health, University College London, London, United Kingdom; 5 Institute of Behavioral Sciences, University of Helsinki, Helsinki, Finland; 6 Institute of Molecular Medicine Finland FIMM, Helsinki, Finland; 7 Department of Chronic Disease Prevention, National Institute for Health and Welfare, Helsinki, Finland; 8 Department of Human Genetics, Wellcome Trust Sanger Institute, Cambridge, United Kingdom; 9 Public Health Consulting, Europe, Helsinki, Finland; 10 Department of Mental Health and Substance Abuse Services, National Institute for Health and Welfare, Helsinki, Finland; 11 Department of Psychiatry, University of Helsinki and Helsinki University Central Hospital, Helsinki, Finland; Innsbruck Medical University, Austria

## Abstract

**Background:**

Psychological stress is suggested to accelerate the rate of biological aging. We investigated whether work-related exhaustion, an indicator of prolonged work stress, is associated with accelerated biological aging, as indicated by shorter leukocyte telomeres, that is, the DNA-protein complexes that cap chromosomal ends in cells.

**Methods:**

We used data from a representative sample of the Finnish working-age population, the Health 2000 Study. Our sample consisted of 2911 men and women aged 30–64. Work-related exhaustion was assessed using the Maslach Burnout Inventory - General Survey. We determined relative leukocyte telomere length using a quantitative real-time polymerase chain reaction (PCR) -based method.

**Results:**

After adjustment for age and sex, individuals with severe exhaustion had leukocyte telomeres on average 0.043 relative units shorter (standard error of the mean 0.016) than those with no exhaustion (p = 0.009). The association between exhaustion and relative telomere length remained significant after additional adjustment for marital and socioeconomic status, smoking, body mass index, and morbidities (adjusted difference 0.044 relative units, standard error of the mean 0.017, p = 0.008).

**Conclusions:**

These data suggest that work-related exhaustion is related to the acceleration of the rate of biological aging. This hypothesis awaits confirmation in a prospective study measuring changes in relative telomere length over time.

## Introduction

Work-related stress occurs when the demands of the work environment exceed the worker’s ability to cope with or control them [1]. In Europe, its prevalence is around 20% and the annual cost of work-related stress is estimated to be 20 billion euro in the 15 original European Union Member States [2]. In the US, workers experiencing fatigue were estimated to cost employers an excess of 101 billion dollars [3]. In addition to financial concerns, chronic stress seems to be detrimental to individual health, increasing the risk of cardiovascular diseases, immune dysfunction, and neuropsychiatric diseases [4]–[7]. Work-related exhaustion is a psychological consequence of prolonged work stress [8]. It coexists at high rates with mental and physical illnesses [9], [10].

Accelerated cellular aging has been proposed as one possible mechanism linking chronic stress to adverse health outcomes [11]. Telomeres, the DNA-protein complexes that cap chromosomal ends in cells, might serve as a biomarker of a cell’s biological age [12]. When cells divide, the telomere is not fully replicated because of the limitations of the DNA polymerase in completing the replication of the ends of the linear molecule, leading to telomere shortening with every replication. Finally, after numerous shortenings, the cell is arrested into senescence.

Leukocyte telomeres have been found to be shorter among older people, smokers, obese individuals, people with low education, and those with care giving stress [11], [13]–[15]. Among 58 healthy mothers, perceived stress was related to leukocyte telomere length and their telomeres were shown to shorten linearly as a function of the years spent taking care of a severely ill child [11]. In addition, childhood adverse life events [16] and lifetime depressive exposure was related to shorter telomeres [17]. However, the extent to which work-related stress is associated with telomere length is so far unknown.

The aim of the present study was to investigate if work-related exhaustion is related to leukocyte telomere length in a large working population. Our data from the Health 2000 Study of Finnish men and women confirmed an association between severe work-related exhaustion and shorter leukocyte telomeres.

## Methods

### Ethics Statement

This study was approved by the Ethics Committee of Epidemiology and Public Health of the Hospital District of Helsinki and Uusimaa. After the subjects received a complete description of the study, we obtained their written informed consent.

### Study Sample

The Health 2000 Study was carried out in Finland between August 2000 and June 2001. The two-stage stratified cluster sample was representative of the Finnish mainland population and included 8028 people aged 30 or over. Stratification and sampling were conducted as follows: The strata were five university hospital districts, each serving about one million inhabitants and differing in several features in relation to geography, economic structure, health services, and the socio-demographic characteristics of the population. First, the 15 largest towns in Finland were included with a probability of one. Next, within each of the five districts, 65 other areas were sampled applying the probability proportional to population size (PPS) method. Finally, from each of these 80 areas, a random sample of individuals was drawn from the National Population Register so that the total number of people drawn from each stratum was proportional to the population size of the area in question [18].

**Figure 1:**
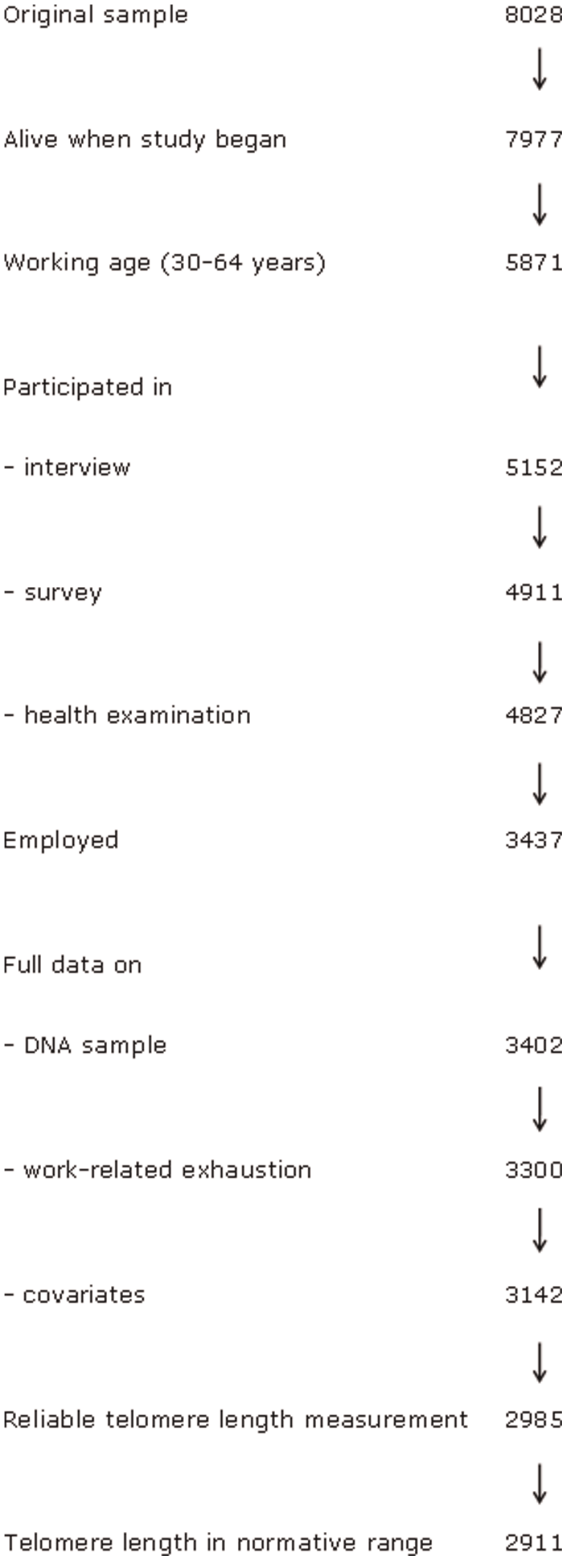
Sample selection.

Figure 1 shows a flow chart of the various study phases. Of the sample (n = 8028), 51 died before the study began. A total of 5871 subjects were of working age (30–64 years). We excluded the subjects who did not participate in the interview (n = 719), return the questionnaire (n = 241), attend the health examination conducted approximately four weeks later (n = 84), report working as their main activity (n = 1390), or who had critical missing data (35 on DNA sample, 102 on work-related exhaustion, 158 on covariates). Due to unreliable telomere length measurement, we excluded 157 subjects. In addition, we excluded 74 subjects due to possible outlier values in telomere length (over 1.7999 relative units). Participants with outlier values in telomere length were somewhat younger than the other participants (39 vs. 45 yrs, p<0.001). There were no other statistically significant differences between the groups of included and excluded participants due to the values of telomere length (p>0.29). The study sample proper consisted of 2911 men and women.

### Telomere Length Measurement

Relative leukocyte telomere length was determined from genomic DNA extracted from peripheral blood by a quantitative real-time PCR-based method [16], [19], [20] with modifications as follows: Amplification signals were quantified by the standard curve method using a DNA template series (0.5, 1.0, 2.5, 5.0, 7.5, 10, and 15 ng) on every plate. The correlation coefficient of the standard curve was on average 0.997 for the telomere reaction (range 0.994 to 0.999) and 0.997 for β-hemoglobin reaction (range 0.993 to 0.999). The corresponding PCR reaction efficiencies were 88.4% for the telomere reaction (range 77.8 to 97.1%) and 92.7% for β-hemoglobin reaction (range 85.9 to 100.4%). Relative telomere length (T/S ratio) was determined by comparing the value of absolute quantification of telomere DNA with a single copy reference gene, β-hemoglobin. All samples were measured as triplicates and the averages were used for further analyses. Samples with standard deviation of greater than 0.5 between the triplicates were omitted (n = 157). Plate effect was taken into account by normalizing the telomere signal and reference gene signal to the corresponding average of five control samples analyzed on every qPCR plate. The five control samples were also used to calculate the coefficient of variation (CV) of the method. This was 7.0% for the telomere reaction, 5.5% for the β-hemoglobin reaction, and 8.1% for their ratio (T/S).

### Work-related Exhaustion

We assessed work-related exhaustion using the Maslach Burnout Inventory – General Survey (MBI-GS) [21]–[23]. MBI-GS has proven to be a reliable and valid measure of occupational burnout, a psychological consequence of prolonged work stress [24]–[25]. The exhaustion subscale consists of five items which are rated on a seven-point frequency rating scale ranging from 0 (never) to 6 (daily). We calculated an averaged summary score which was then categorized as follows: no exhaustion (0–1.49), mild exhaustion (1.50 - 3.49), and severe exhaustion (3.50–6). According to this categorization, in severe exhaustion the symptoms were experienced approximately daily or weekly, in mild exhaustion monthly, and in cases of no exhaustion only a few times a year or never [22], [26].

### Covariates

#### Physical illness

The participants’ physical symptoms and known diseases were elicited at the beginning of the health examination. After the measurements, the research physician collected anamnestic data and performed a standard 30-minute clinical examination. The diagnostic criteria of the physical illnesses were based on current clinical practice. The participant was identified as a case (yes/no) if the diagnostic criteria for one or more physical illness (e.g. musculoskeletal disorders, cardiovascular diseases, respiratory diseases, or other physical illnesses) were fulfilled [27].

#### Common mental disorders

Common mental disorders were assessed using the computerized version of the Composite International Diagnostic Interview (M-CIDI) in the health examination [28]. The M-CIDI was carried out by health care workers who were trained for the interview by psychiatrists and physicians who had themselves been trained by a WHO authorized trainer. The program uses operationalized criteria for diagnoses in the Diagnostic and Statistical Manual of Mental Disorders (DSM-IV) [29] and enables the estimation of DSM-IV diagnoses for major mental disorders. The variable of a common mental disorder (yes/no) included depressive disorders (major depressive disorder and dysthymic disorder), anxiety disorders (panic disorder either with or without agoraphobia, generalized anxiety disorder, social phobia not otherwise specified, and agoraphobia without panic disorder), and alcohol use disorders (alcohol dependence and alcohol abuse).

#### Health behavior

Smoking and body mass index were assessed as telomere length-related aspects of health behavior. We assessed current daily smoking (yes/no) in the interview. Body mass index (kg/m^2^) was calculated from the clinical measurements taken in the health examination.

#### Sociodemographic factors

In the interview we collected information on sex, age (yrs), marital status, occupation, and type of business. Marital status was divided into groups of married (married or cohabiting) and unmarried (single, widowed, or divorced). Occupational grade (manual, lower non-manual, higher non-manual, self-employed) was formed on the basis of occupation and type of business [30].

### Statistical Analysis

The relative telomere length was normally distributed. The associations between the level of exhaustion and the covariates were described by cross-tabulations and χ ^2^ tests for categorical variables, and by linear regression analysis for continuous variables. The associations between telomere length and the covariates were investigated using linear regression models. We then used linear regression analysis to investigate the sex and age-adjusted association between exhaustion, categorized in three classes, and telomere length. Next, we adjusted this model for marital status, occupational grade, daily smoking, body mass index, physical illness, and common mental disorders.

A complex sampling design, such as ours, can influence variance estimates of both population averages and regression coefficients [31]. Therefore, we used weighting adjustment and sampling parameters in the analyses. Weighting included adjustment for sampling, region, and age, gender, and mother tongue on the participants. This procedure allowed the translation of sample data into population averages, as expressed by weighted percentages, means, and beta-coefficients. This helps to account for the survey design, clustering in a stratified sample, and the loss of participants. The data were analyzed using the Sudaan 9.0.1 statistical program package [32], which is specifically designed for analyzing cluster-correlated data in complex sample surveys.

## Results

### Characteristics of Study Participants

The characteristics of 2911 study participants are presented in Table 1. Fifty-three percent were men, 78% were married or cohabiting, and 29% were manual workers. The mean age of the participants was 45 years. A quarter of them smoked daily and, on average, the participants were slightly overweight. Fifty-seven percent had a physical illness and 13% fulfilled the criteria for a common mental disorder (12 months’ prevalence).

**Table 1:** Characteristics of the study population (N = 2911).

	N (weighted %)	Mean (SE)
Sex		
Women	1445 (47)	
Men	1466 (53)	
Marital status		
Unmarried	639 (22)	
Married	2272 (78)	
Occupational grade		
Manual	834 (29)	
Lower nonmanual	813 (27)	
Upper nonmanual	819 (28)	
Self-employed	445 (16)	
Daily smoking		
No	2186 (75)	
Yes	725 (25)	
Somatic illness		
No	1273 (43)	
Yes	1638 (57)	
Mental illness		
No	2531 (87)	
Yes	380 (13)	
Age		44.8 (0.15)
Body mass index		26.4 (0.07)

Mild work-related exhaustion was experienced by 18% and severe work-related exhaustion by 5%. Work-related exhaustion was related to sex, age, marital status, occupational grade, and having a physical illness or a common mental disorder (Table 2). Severe exhaustion was more common among female, unmarried, manual, and ill workers. Workers with severe exhaustion (mean 47 yrs, SE = 0.72) or with mild exhaustion (mean 46 yrs, SE = 0.38) were also older than workers with no exhaustion (mean 44 yrs, SE = 0.18; F = 9.37, p<0.001). Body mass index was not related to exhaustion (F = 1.34, p = 0.263).

**Table 2:** Level of work-related exhaustion by characteristics of the study population.

	Exhaustion			Statistics
	No	Mild	Severe	
	N (%^a^)	N (%^a^)	N (%^a^)	X^2^, p
All	2229 (77)	530 (18)	152 (5)	
Sex				10.4, 0.001
Women	1054 (73)	301 (21)	90 (6)	
Men	1175 (80)	229 (16)	62 (4)	
Marital status				4.33, 0.013
Unmarried	460 (72)	137 (21)	42 (7)	
Married	1769 (78)	393 (17)	110 (5)	
Occupational grade				2.93, 0.008
Manual	608 (73)	170 (20)	56 (7)	
Lower nonmanual	659 (81)	118 (14)	36 (5)	
Upper nonmanual	617 (76)	160 (19)	42 (5)	
Self-employed	345 (77)	82 (18)	18 (4)	
Daily smoking				0.12, 0.891
No	1678 (77)	394 (18)	114 (5)	
Yes	551 (76)	136 (19)	38 (5)	
Somatic illness				15.2, 0.001
No	1031 (81)	198 (15)	44 (3)	
Yes	1198 (73)	332 (20)	108 (7)	
Mental illness				56.8, 0.001
No	2036 (81)	408 (16)	87 (3)	
Yes	193 (51)	122 (32)	65 (17)	

a Weighted percentage.

### Associations with Relative Leukocyte Telomere Length

**Figure 2:**
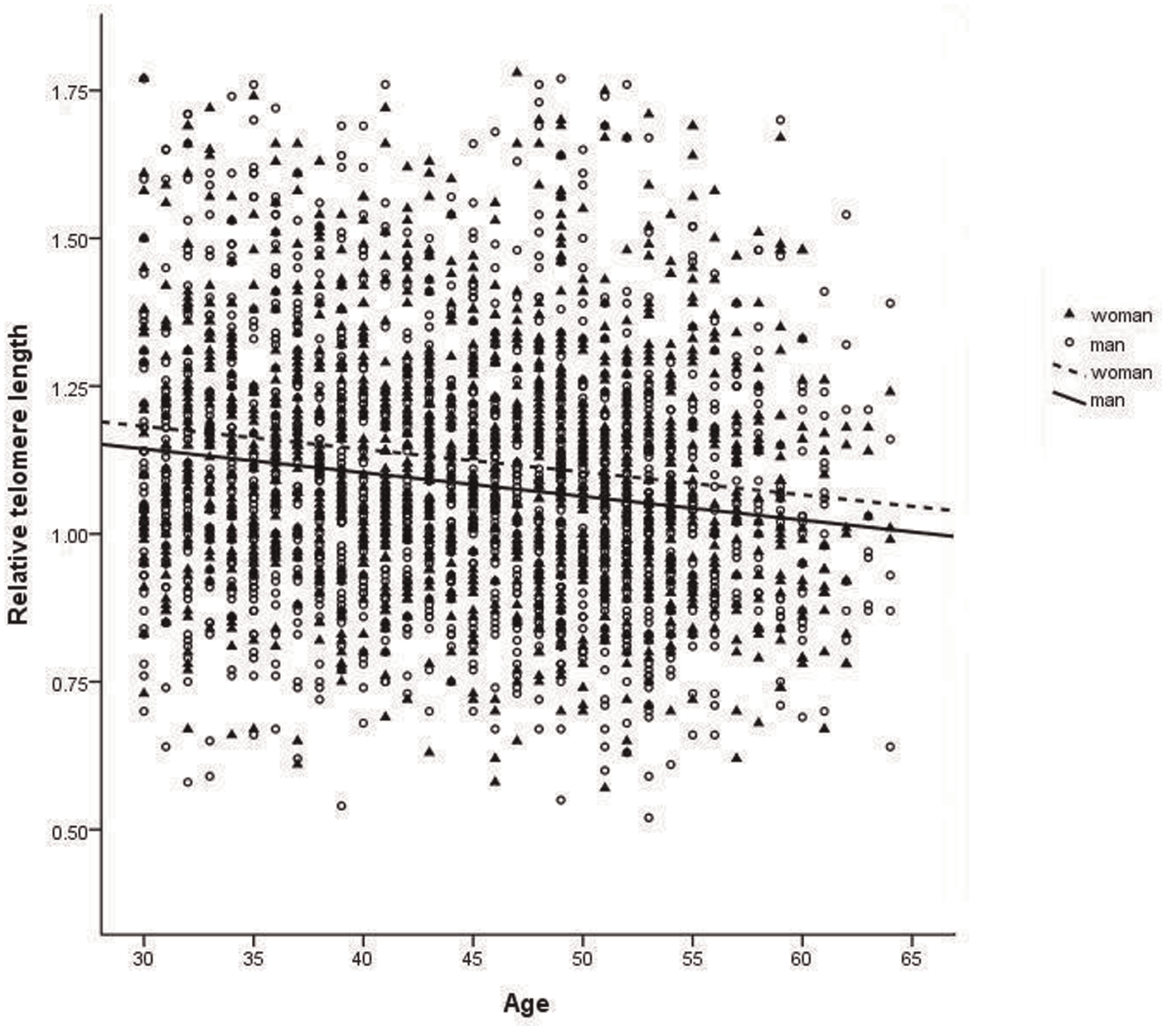
Association between age and relative leukocyte telomere length by sex.

Relative leukocyte telomere length (T/S ratio) averaged 1.104 (SE 0.005). Age was positively related to relative telomere length (Figure 2). In addition, sex and body mass index were related to relative telomere length. Male sex and higher body mass index were associated with shorter telomeres (Table 3). Telomere length was not related to marital status, occupational grade, smoking, or having a physical illness or a common mental disorder.

**Table 3:** Association between relative leukocyte telomere length and the covariates.

Covariate		β	SE	p-value	F	p-value
Sex					28.6	0.001
	Women	ref.				
	Men	−0.037	0.007	0.001		
Age		−0.004	0.001	0.001	60.2	0.001
Marital status					0.01	0.936
	Unmarried	ref.				
	Married	0.001	0.010	0.936		
Occupational grade					0.01	0.999
	Manual	ref.				
	Lower nonmanual	0.001	0.011	0.893		
	Upper nonmanual	0.001	0.011	0.899		
	Self-employed	0.002	0.013	0.887		
Daily smoking					0.29	0.589
	No	ref.				
	Yes	0.005	0.009	0.589		
Body mass index		−0.003	0.001	0.001	14.3	0.001
Somatic illness					0.12	0.732
	No	ref.				
	Yes	−0.003	0.009	0.732		
Mental illness					0.58	0.445
	No	ref.				
	Yes	−0.009	0.012	0.445		

**Figure 3:**
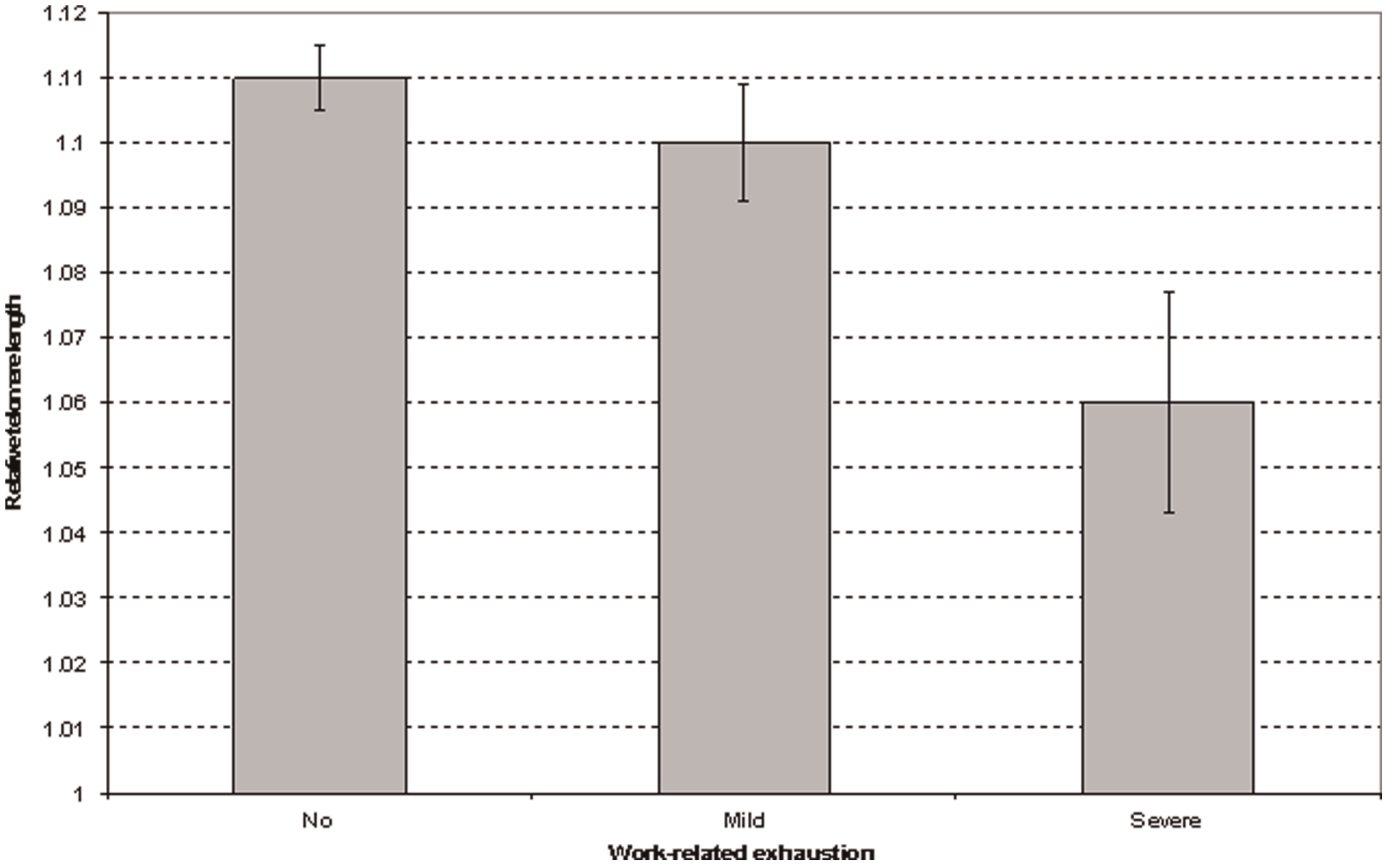
Relative leukocyte telomere length by work-related exhaustion. Error bars represent standard error of mean.

Sex and age-adjusted relative telomere length was related to work-related exhaustion (Table 4). Those with severe work-related exhaustion had shorter telomeres than those with mild or no exhaustion (Figure 3). The difference in telomere length was on average 0.043 relative units between participants with severe exhaustion and those with no exhaustion and 0.002 relative units between those with mild exhaustion and those with no exhaustion. The association between work-related exhaustion and relative telomere length remained statistically significant after adjustment for marital status, occupational grade, daily smoking, body mass index, physical illness, and common mental disorders (Table 4). After adjustments, the difference was 0.044 relative units between those with severe exhaustion and those with no exhaustion and 0.003 between those with mild exhaustion and those with no exhaustion.

**Table 4:** Association between level of work-related exhaustion and relative leukocyte telomere length.

		β	SE	p-value	F	p-value
Model 1^a^	Exhaustion				3.48	0.031
	No	ref.				
	Mild	−0.002	0.010	0.851		
	Severe	−0.043	0.016	0.009		
Model 2^b^	Exhaustion				3.49	0.031
	No	ref.				
	Mild	−0.003	0.010	0.797		
	Severe	−0.044	0.017	0.008		

a Model 1 is adjusted for sex and age.

b Model 2 is adjusted for sex, age, marital status, occupational grade, daily smoking, body mass index, physical illness, and common mental disorders.

## Discussion

To our knowledge, this is the first study to show that relative leukocyte telomere length is related to work-related exhaustion, a marker of prolonged environmental stress in the work context. In our population-based sample of almost 3000 adults, severe work-related exhaustion was related to shorter leukocyte telomeres. Our findings were not explained by other factors such as sociodemographic factors, smoking, body mass index, physical illness, or common mental disorders.

Due to the cross-sectional design of our study, the blood samples for the leukocyte telomere length measurement were collected in parallel with the assessment of work-related exhaustion. Therefore it was not yet possible to prospectively analyze the shortening of the telomeres along with increasing exhaustion. In addition, we had no data on the duration of exhaustion. In previous studies, telomere shortening has been more consistently related to prolonged or accumulated psychosocial stress or illness than to acute stress or illness [11], [14], [16], [17]. In the present study, the level of exhaustion may be regarded as a crude estimate for the duration of work-related exhaustion because work-related exhaustion accumulates over several years of mismatch between the demands and resources of one’s job [33]. In accordance with earlier studies on psychosocial stress [11], [14], [16], [17], a mild level of work-related exhaustion was not related to telomere length in the present study. Furthermore, we were able to adjust for common mental disorders (i.e., depressive, anxiety, and alcohol use disorders) which are quite prevalent among the working-age population (12 moths’ prevalence as 4–6%) [34]. Instead, we were not able to take into account the effect of severe, for example psychotic, mental disorders. We also adjusted for physical illnesses regardless of their severity.

Although one of the strengths of our study is the population-based epidemiological sample that represents all the working age people living in mainland Finland, it comes with the limitation regarding the available DNA amount. We measured relative leukocyte telomere length using qPCR-based method, in which a smaller amount of DNA is needed compared to the Southern blot method. The qPCR-based method has been used in epidemiological settings and it was shown to produce comparable results to the Southern blot method [35]. However, the qPCR method is not able to detect the distribution of the individual telomere length or the length of the shortest telomere that may be more informative measures biologically.

Our assessment of cellular aging was limited to leukocyte telomere length. However, the synthesis and maintenance of telomeres is a complex process requiring a specialized reverse transcriptase (telomerase) that adds specific G-rich nucleotide repeats onto the 3′ ends of telomeric DNA. In the absence of telomerase or when this enzyme is expressed at very low levels, DNA synthesis during cell division results in the progressive shortening of telomeric DNA. This erosion eventually compromises telomere integrity, triggering a DNA damage response which in turn results in the onset of senescence [36]. Future studies on work-related exhaustion should therefore measure telomerase activity in addition to telomere length.

Prolonged work stress is believed to affect health via “the wear and tear of the organism” [6]. Telomere shortening has been suggested as a part of a general stress response in various situations which are chronically demanding to the organism [12], [13], [37]. The present results support the idea that part of the “tearing” in work stress situations might result from accelerated cellular aging.

In line with previous studies, relative leukocyte telomere length was also related to sex, age, and body mass index in our sample [11], [13]–[15]. Men, older people, and obese people had shorter telomeres than the others. Those excluded from the present study on the basis of possible outlier values in telomere length were 6 years younger than those included. We used a conservative threshold for removing outlier samples. Therefore we might have removed some young individuals with truly exceptionally long telomeres. However, since they did not differ from those included regarding work-related exhaustion, it is unlikely that the exclusion of these people would have affected our conclusions.

Contrary to previous studies, smoking was not related to leukocyte telomere length in this sample [11], [13]–[15]. Smoking is a state of heightened oxidative stress which increases the rate of telomere erosion per replication. A dose-dependent relationship between the amount of pack-years smoked and telomere length has been demonstrated [11]. We used a dichotomized variable of current daily smoking in the present study. It did not take into account the amount of tobacco smoked or the length of the smoking history. This limitation may explain the lack of association between smoking and telomere length in this study. Therefore, the smoking variable used may be too crude a measure to capture the effect of smoking on telomere length.

In conclusion, work-related exhaustion may relate to the rate of acceleration of biological aging. This hypothesis awaits confirmation in a prospective study measuring changes in relative telomere length over time. Further research is also needed to determine whether reduced leukocyte telomere length, as a potential cellular phenotype, underlies the association between prolonged work stress and an increased risk of degenerative diseases, such as coronary heart disease and stroke.
